# Recent Trends in Multifunctional Liposomal Nanocarriers for Enhanced Tumor Targeting

**DOI:** 10.1155/2013/705265

**Published:** 2013-03-07

**Authors:** Federico Perche, Vladimir P. Torchilin

**Affiliations:** ^1^Center for Pharmaceutical Biotechnology and Nanomedicine, Northeastern University, 140 the Fenway, Room 236, 360 Huntington Avenue, Boston, MA 02115, USA; ^2^Center for Pharmaceutical Biotechnology and Nanomedicine, Northeastern University, 140 the Fenway, Room 214, 360 Huntington Avenue, Boston, MA 02115, USA

## Abstract

Liposomes are delivery systems that have been used to formulate a vast variety of therapeutic and imaging agents for the past several decades. They have significant advantages over their free forms in terms of pharmacokinetics, sensitivity for cancer diagnosis and therapeutic efficacy. The multifactorial nature of cancer and the complex physiology of the tumor microenvironment require the development of multifunctional nanocarriers. Multifunctional liposomal nanocarriers should combine long blood circulation to improve pharmacokinetics of the loaded agent and selective distribution to the tumor lesion relative to healthy tissues, remote-controlled or tumor stimuli-sensitive extravasation from blood at the tumor's vicinity, internalization motifs to move from tumor bounds and/or tumor intercellular space to the cytoplasm of cancer cells for effective tumor cell killing. This review will focus on current strategies used for cancer detection and therapy using liposomes with special attention to combination therapies.

## 1. Introduction

**Figure 1:**
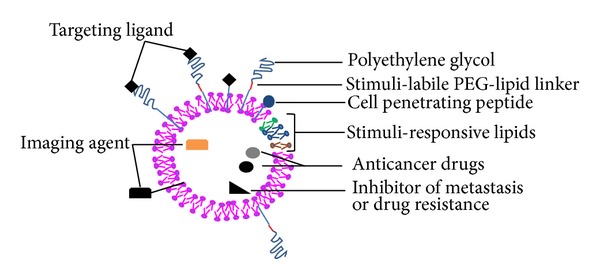
Schematic picture of a multifunctional liposomal nanocarrier.

**Figure 2:**
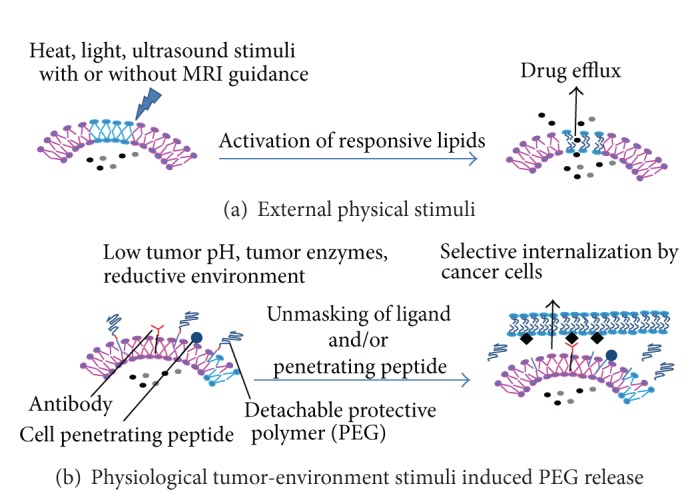
Schemes for tumor-specific liposome destabilization or endocytosis.

**Figure 3:**
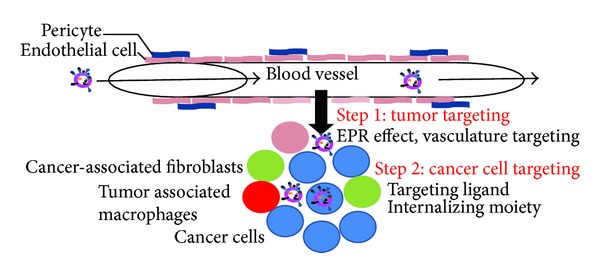
Targeting mechanisms in liposomal cancer therapy.

Liposomes, first described in 1965 [1, 2], are established drug and gene delivery carriers with clinical evidence of efficacy [3–5] and several commercially available approved clinical formulations [6]. Liposomes are lipid vesicles either unilamellar or multilamellar with an aqueous compartment. The structure of liposomes allows for delivery of a cargo loaded in the aqueous compartment or embedded in the lipid bilayer for cancer therapy, noninvasive cancer imaging, or therapy [7, 8]. As recently reviewed [9], the most important property of liposomal nanocarriers is protection from the degradation and optimization of the pharmacokinetics of the encapsulated drug to improve tumor accumulation and therapeutic efficacy while reducing the adverse effects associated with bolus administration [7, 10, 11]. This paper will focus on the use of liposomal nanocarriers in cancer therapy and diagnosis. Cancer therapy targets the hallmark traits of cancer: deregulated cell growth, evasion from apoptosis, sustained angiogenesis, tissue, invasion and metastasis [12]. Liposomes remain one of the first drug delivery carrier tested for improvement of pharmacokinetics of new anticancer drugs with more than 2000 papers and 200 reviews published in 2011 and many liposomal drugs approved for cancer therapy notably Doxil for doxorubicin (Johnson & Johnson, New Brunswick, USA), Lipusu for paclitaxel (Luye Pharma Group, Yantai, China), and Marqibo for vincristine (Talon Therapeutics, South San Francisco, USA) [7, 13–15]. The liposomal platform has undergone continuous optimization for improved stability *in vivo*, high drug and/or imaging agent loading, stimuli-targeted delivery of the cargo at the tumor site for efficient uptake by cancer cells, and intracellular payload release to engineer multifunctional liposomal nanocarriers (Table 1, Figures 1–3) [16]. We will describe the main axes of design of multifunctional liposomal nanocarriers.

## 2. Stealth Targeted Liposomes

### 2.1. Stealth Liposomes

Effective cancer treatment generally implies drug delivery to cancer cells after systemic administration by taking advantage of the leaky tumor vasculature to deposit at the tumor site [17]. Indeed, liposome uptake by tumors relies primarily on the enhanced permeability and retention (EPR) effect [13, 17–19]. EPR is dependent on large endothelial fenestrations in the tumor endothelial vasculature coupled with the incomplete pericyte coverage that permits extravasation of large molecules and liposomes of size below 200 nm into tumors with an impaired lymphatic drainage that is responsible for their retention [17, 18, 20]. However, after parenteral administration, most liposomes are captured by the mononuclear phagocyte system (MPS) in the liver and spleen [21]. This elimination is due to the recognition by serum proteins (opsonins) and complement components which prime liposomes for macrophage removal from the circulation [21, 22]. The step, required to increase the probability of extravasation at the tumor site, involves extended stabilization, decreased blood clearance, and capture by the MPS to favor their accumulation in tumors (Figure 2) [7, 8, 23]. 

To achieve this, two approaches are currently used in preclinical and clinical liposomal drug carriers [44]. Decrease of membrane fluidity through incorporation of cholesterol to impede lipid extraction by high density lipoproteins in the blood associated with to liposome breakdown (approved formulations DaunoXome, Myocet, Depocyt, Mariqibo, Doxil) [44, 45]. The second approach is the incorporation of flexible hydrophilic moieties, mainly polyethylene glycol(PEG), since this component is approved for use by the United States Food and Drug Administration and is currently used in several approved formulations (Doxil, SPI-077, S-CDK602) [7, 10, 44, 46], but also polyvinyl pyrrolidones [8] or Poly[N-(2-hydroxypropyl)methacrylamide] [47]. The inclusion of flexible hydrophobic inert and biocompatible polyethylene glycol, (PEG) with a lipid anchor in liposome allows the formation of an hydrated steric barrier decreasing liposome interaction with blood-borne component, increasing their blood circulation time, decreasing their spleen and liver capture [48, 49], and their resistance to serum degradation [50]. This lack of recognition by the MPS and decreased elimination of PEGylated liposomes led to the term “stealth” liposomes to qualify them [44]. 

Protection by PEG was shown to be dependent on both the PEG molecular weight and density on the liposome surface with ~5% by weight, allowing the maximal decrease in protein adsorption and enhanced blood circulation time [51]. Longer blood circulation time, decreased spleen and liver capture, and increased tumor accumulation after intravenous injection have been reported for ^111^In-labeled liposomes containing 6% PEG compared to 0.9% PEG [52]. Lee et al. compared the liver and spleen accumulation of ^99m^Tc-labeled liposomes containing 0, 5, 9.6, or 13.7% PEG (molar ratio) [53]. While 5 or 9.6% PEG decreased spleen and liver accumulation compared to unPEGylated liposomes, spleen accumulation increased again with 13.7% PEG, indicating an upper limit to the effect of PEGylation. When PEG chains of different lengths were appended to the surface of immunoliposomes, as short (750 Da), intermediate (2000 Da), or long PEG (5000 Da), DSPE-PEG2000 was the best compromise for extended blood circulation and target binding *in vivo.* PEG750 did not improve blood circulation and PEG5000 decreased ligand binding [54]. 

Similarly, superior interaction of cell penetrating peptide-modified PEGylated liposomes with cells was evidenced *in vitro *after coupling of the peptide to PEG1000 over PEG750 or PEG3400 and was correlated with the architecture of ligand presentation [55]. The longer blood residency of PEGylated liposomes associated with their lower elimination by the MPS has been correlated with increased tumor accumulation and efficacy [19, 21, 23, 56]. However, liver, spleen, and bone marrow remain the final destinations of empty or drug-loaded PEGylated liposomes [23, 56]. Improvement of drug pharmacokinetics and therapeutic efficacy after encapsulation in PEGylated liposomes was well illustrated by Yang et al. [57]. Indeed, PEGylation of paclitaxel-loaded liposomes led to increased plasma and tumor levels of paclitaxel, in parallel decreased liver and spleen paclitaxel levels over Taxol or conventional paclitaxel liposomes and resulted in the best tumor growth inhibition [57]. 

Interestingly, albumin conjugation to drug-loaded PEGylated liposomes further enhanced their circulation time and resulting therapeutic activity [58, 59]. Indeed the blood clearance of doxorubicin after intravenous administration in rats decreased from 131 mL/h for free doxorubicin to 17.9 mL/h for PEGylated liposomal doxorubicin and decreased further to 7 mL/h for PEGylated and albumin-conjugated doxorubicin-loaded liposomes. Albumin also decreased opsonin binding to PEGylated liposomes and improved the therapeutic activity of doxorubicin-loaded liposomes against sarcoma. 

Inclusion of PEG in the liposome is achieved either by mixing a lipid-anchored PEG with the liposome forming lipids prior to liposome formation (preinsertion) or by insertion of PEG-lipid in already formed liposomes (postinsertion). These two approaches are currently used in clinically approved formulations [44]. Postinsertion of DSPE-PEG2000 compared to its preinsertion in irinotecan-loaded liposomes revealed higher plasma concentration and slower drug release in rats [60]. Of note, this longer blood circulation time was correlated with better therapeutic efficacy of postinserted DSPE-PEG2000 drug-loaded liposomes. Although the lipid-PEG conjugates can be incorporated in liposomes before their formation (preinsertion) or inserted into preformed liposomes, the former strategy induces presentation of the PEG groups both at the liposomal surface and in reverse orientation at the inner side of the lipid bilayer. This results in decreased drug loading and stealth properties of the liposomes. Indeed, when both strategies of PEGylation were compared, higher blood circulation and higher therapeutic efficacy *in vivo* of postinsertion over preinsertion modification were demonstrated [60, 61].

A new alternative to increase the circulation time of drug-loaded liposomes is the use of superhydrophilic zwitterionic polymers to create a hydrated shell around the liposome [62]. Cao et al. compared the therapeutic activity of two doxorubicin formulations, Doxil where DSPE-PEG2000 imparts blood stability and doxorubicin-loaded liposomes containing the zwitterionic lipid DSPE-poly(carboxybetaine) for the same function. Similar doxorubicin accumulation in tumors after intravenous administration was detected for both formulations, but poly(carboxybetaine) containing liposomes led to an earlier cure of tumor-bearing mice validating this chemistry.

#### 2.1.1. Importance of Charge Neutralization for Passive Targeting

Although neutral non-PEGylated radiolabeled liposomes were shown to accumulate in human tumors [63], PEGylation is required for effective tumor localization. PEGylation protected against aggregation of assemblies made with cationic lipids, enhanced their tumor uptake, and decreased their accumulation in the liver [64]. Campbell et al. compared the biodistribution of negatively charged liposomes (−20 mV) and positively charged liposomes (+31 mV) after intravenous injection to tumor-bearing mice [65]. While liver was the major destination for both formulations with more than 50% of the injected dose, positively charged liposomes showed lower spleen accumulation and higher lung accumulation. Interestingly, in tumors, positively charged liposomes showed higher association with tumor blood vessels than negatively charged ones. Levchenko et al. proposed the modulation of positively and negatively charged liposomes biodistribution by different opsonins [66]. Moreover, neutral PEGylated liposomes encapsulating doxorubicin showed superior therapeutic activity compared to cationic ones the decreased antitumor efficacy was correlated with reduced blood circulation and tumor accumulation of cationic liposomes [67]. A critical correlation between negative liposome charge and uptake by liver and spleen has been reported [66]; charge shielding by PEG decreased liver uptake and prolonged blood circulation. Finally, Huang and coworkers reported abolishment of liver uptake of cationic liposomes after their neutralization by postinsertion of DSPE-PEG leading to an increased tumor accumulation [68].

#### 2.1.2. Importance of Prior Administration/Accelerated Blood Clearance (ABC)

Cancer treatments usually imply repeated administration of the same therapeutic agent to previously treated (predosed) patients. Administration of radiolabeled PEGylated liposomes to animals pretreated with a first dose of PEGylated liposomes revealed a drastic decrease of their blood concentration 4 h after injection from 50% of the injected dose for naive animals to 0.6% of the injected dose for predosed animals [69]. Noteworthy, after the second administration, PEGylated liposomes were cleared from the circulation very rapidly (decrease in half-life from 2.4 h to 0.1 h) and this decreased blood residency was mirrored by increased accumulation in liver and spleen, supporting the accelerated blood clearance of liposomes after their second administration. This phenomenon is termed accelerated blood clearance (ABC). ABC is dependent on the time after initial injection: no ABC was reported for PEGylated liposomes injected daily or with injection intervals less than 5 days in rats whereas a one week interval induced accelerated blood clearance in the same study [69]. This delay reflects the two phases of ABC [70, 71]. First, anti-PEG IgM is secreted in the spleen during the effectuation phase [72, 73], an organ where both drug-loaded PEGylated and non-PEGylated liposomes accumulate [23, 74]. Second, during the effectuation phase, opsonisation of PEGylated liposomes by anti-PEG IgM primes them for elimination by liver macrophages [75]. Tagami et al. recently demonstrated that production of anti-PEG2000-DSPE IgM in mouse after administration of PEGylated lipoplexes was higher with PEGylated liposomes harboring siRNA on their surface over PEGylated liposome-wrapped siRNA lipoplexes [76]. Moreover, the same group reported higher anti-PEG IgM production after parenteral injection of PEGylated DNA lipoplexes prepared with adjuvant CpG motifs-containing pDNA over PEGylated lipoplexes prepared with pDNA devoid of CpG motifs [77]. This lower anti-PEG IgM production from CpG-free lipoplexes was correlated with lower accelerated blood clearance. Both of these studies suggest an important effect of the liposome cargo in anti-PEG IgM production and the ABC phenomenon. 

Anti-PEG IgM production is not limited to PEGylated liposomes; anti-PEG IgM was also detected in rats injected with PEGylated adenovirus, bovine serum albumin, or ovalbumin [78]. Interestingly, Laverman et al. reported no ABC induction of Doxil when rats were preinjected with Doxil one week before administration, whereas preinjection with empty PEGylated liposomes induced ABC of Doxil [70]. These data suggest prevention of ABC by doxorubicin entrapment in liposomes. This has been attributed to a decreased clearance capacity of Doxil-injected rats due to toxicity of doxorubicin for liver macrophages [79]. By contrast, Van Etten et al. reported no decrease in bacterial clearance after Doxil injection [80] suggesting a macrophage-independent mechanism. Kiwada and coworkers reported the induction of anti-PEG IgM production in the spleen after administration of PEGylated liposomes priming them for elimination by liver macrophages and also demonstrated decreased ABC in splenectomized rats which was correlated with lower anti-PEG IgM titers [72]. 

Longer blood circulation of doxorubicin-loaded PEGylated liposomes after a second administration has been observed in mice, dogs, rats, and patients [70, 81–83] and was proposed to be due to toxicity towards splenic B cells [70]. The importance of toxicity in resistance to ABC by Doxil liposomes is supported by the suppression of IgM production after a second administration of oxaliplatin-loaded PEGylated liposomes compared to empty PEGylated liposomes [84] and by the evidence of ABC induction with PEGylated topotecan-loaded liposomes that have a fast drug release rate [85]. Additionally, blood clearance of radiolabeled liposomes was inhibited by a preadministration of Doxil whereas preinjection of free doxorubicin or empty liposomes did not inhibit blood clearance [82] further supporting inhibition of the MPS as the mechanism of decreased blood clearance of drug-loaded liposomes. 

However, as pointed out recently by Suzuki et al., there is no report yet of ABC in patients [86] although PEGylated liposomes such as Doxil have been in clinical use for more than 20 years suggesting caution in interpretation of the preclinical model data [86]. Indeed, Gabizon et al. recently reported decreased blood clearance of Doxil after repeated administration in cancer patients [81]. The high variability of pharmacokinetics of drug-loaded PEGylated liposomes in cancer patients [87] should also be considered as it may render an ABC phenomenon difficult to detect without a very large cohort. Although complement activation by PEGylated drug-loaded liposomes has been reported both in animal models and in patients (reviewed in [88]), its correlation with accelerated blood clearance is still controversial [89]. Finally, ABC could be decreased after methylation of the anionic charge on the phosphate group of PEG [90] further improving pharmacokinetics of PEGylated liposomes. 

### 2.2. Targeted Stealth Liposomes

As recently reviewed, PEGylation fails to lead to more than 5% of the administered formulation accumulation in the tumor [23, 91]. Furthermore, although radiolabeled liposomes were shown to accumulate in solid tumors in patients, they also distributed to normal organs, revealing the need for tumor targeting [63]. Moreover, most macromolecules, free drugs, and liposomes without an internalization moiety have an accumulation limited to the periphery of a tumor due to the poor vascular density in tumors and the high tumor interstitial fluid pressure impeding transport of macromolecules [92–94]. In a direct comparison of doxorubicin-loaded PEGylated and non-PEGylated liposomes, PEGylation did not improve doxorubicin accumulation in tumors, with comparable therapeutic efficacy of PEGylated and non-PEGylated doxorubicin-loaded liposomes [95]. On the contrary, conjugation of internalizing antibodies with the surface of doxorubicin-loaded PEGylated liposomes dramatically improved their therapeutic efficacy [96, 97] demonstrating the need for improved internalization of antineoplastic agents for effective therapy [98]. Similarly, while Bartlett et al. reported identical tumor distribution of untargeted and transferrin-targeted siRNA nanoparticles, the latter achieved superior *in vivo* silencing [99]. 

To increase liposomal drug accumulation in the cancer cells, liposomes must combine small size and long circulation to reach the tumor (tumor site targeting), a targeting ligand to discriminate between cancer cells and supportive cells (cancer cell targeting), and an internalizing moiety for intracellular delivery (Figure 3, Table 2). For a combination of long blood circulation and targeting, the ligand must be accessible to the target for recognition while the liposomal surface should be coated with PEG for long blood circulation [117] (Figure 1). Thus, in addition to protection from steric hindrance of the liposome surface by the PEG chains, presentation of the ligand at the distal end of PEG allows better ligand recognition [117, 118] and multivalent binding thanks to the flexibility of PEG [119]. Such a combination allowed ultimately superior therapeutic activity compared to PEGylated drug-loaded liposomes without ligand [32–34, 118, 120, 121]. The rationale of targeting plus PEGylation for antitumor efficacy has been well demonstrated by Yamada et al. using folate-linked PEGylated liposomal doxorubicin [122]. They compared the *in vitro* cytotoxicity and *in vivo* antitumor efficacy of untargeted PEGylated doxorubicin-loaded liposomes, non-PEGylated liposomes harboring folate, and PEGylated liposomes with folate exposure at the liposomal surface. While the non-PEGylated folate-modified liposomes showed the highest toxicity *in vitro*, the highest antitumor efficacy was reported with PEGylated, folate-modified doxorubicin-loaded liposomes. The need for targeted drug delivery for the best antitumor efficacy is not limited to liposomes. Indeed, when Saad et al. compared the therapeutic efficacy of targeted or untargeted paclitaxel delivery using a linear polymer, dendrimer or PEGylated liposomes, the best tumor accumulation and tumor suppression were obtained with targeted delivery systems over untargeted ones and free paclitaxel for the three types of carriers [107]. In agreement with this study, addition of a targeting moiety to PEGylated liposomes containing the near infrared probe NIR-797 or ^111^In improved tumor accumulation of the imaging agent, suggesting the benefit of targeting stealth liposomes for cancer therapy and monitoring [123]. Several ligands, including antibodies and peptides directed against molecular markers of tumor cells or their supportive endothelial cells present in the tumor microenvironment, have been employed for targeted drug delivery [124] (Table 2).

#### 2.2.1. Antibody-Targeted PEGylated Liposomes

Targeted liposomes are obtained either by incorporation of ligand-lipid conjugates during liposome preparation, incorporation of lipids with reactive groups during liposome preparation and subsequent ligand coupling, and finally by insertion of ligand-lipid conjugates into preformed liposomes (postinsertion) [125, 126]. For a comparison of techniques available for antibody conjugation to liposomes we refer the reader to recent reviews [97, 127].

Coupling of the humanized anti-CD22 antibody targeting the lymphocyte marker CD22 to PEGylated doxorubicin-loaded liposomes increased doxorubicin accumulation in Non-Hodgkin's Lymphoma xenografts and increased survival over untargeted doxorubicin-loaded liposomes [33]. The p185HER2 (human epidermal growth factor receptor 2) receptor is upregulated in human cancers of several histology (breast, ovarian, and prostate) with a low basal expression in normal tissues allows cancer-specific delivery with HER2 monoclonal antibody conjugation [128, 129]. Conjugation of a single-chain fragment antibody against HER2 to doxorubicin-loaded liposomes led to higher doxorubicin accumulation in breast cancer xenografts and better tumor control than untargeted PEGylated doxorubicin-loaded liposomes [100]. Conjugation of the recombinant humanized anti-HER2 antibody Herceptin (Genentech, San Francisco, CA, USA) to paclitaxel-loaded PEGylated liposomes also increased drug accumulation in tumors and therapeutic efficacy over untargeted paclitaxel-loaded liposomes [34]. The potentiation of paclitaxel-loaded liposomes by HER2 antibody was due to enhanced drug uptake by receptor-mediated endocytosis since a similar tissue distribution and antitumor activity were reported against breast xenografts expressing low levels of HER2. Indeed, in a seminal study, Kirpotin et al. demonstrated that although HER2 antibody-targeted liposomes and untargeted liposomes had similar accumulation profiles in tumors after intravenous injection, they showed, by flow cytometry and histological analysis of disaggregated tumors, a 5.9-fold higher cancer cell accumulation of immunoliposomes versus untargeted liposomes [98]. Antinuclear autoantibodies are present in both healthy elderly individuals and cancer patients [32]. One of these antibodies, 2C5 monoclonal antibody recognizing cell surface-bound nucleosomes specifically recognizes multiple tumor cell lines [32]. Liposomes conjugated with 2C5 antibody at the distal end of PEG3400-DSPE were preferentially accumulated in tumors [32, 130] and increased the therapeutic activity of doxorubicin-loaded (Doxil) liposomes [102]. Tumor targeting of doxorubicin-loaded liposomes with the Fab' fragment of an anti-MT1-MMP (membrane type 1 matrix metalloproteinase, expressed by cancer cells and endothelial cells) led to increased liposome uptake *in vitro* and higher therapeutic activity *in vivo* [120]. It is noteworthy that, although the tumor accumulation of targeted and untargeted liposomes was similar, the MT1-MMP-targeted doxorubicin-loaded liposomes showed superior tumor protection thanks to enhanced uptake of the drug by tumor cells, in agreement with the results of Kirpotin et al. with anti-HER2 targeted liposomes [98].

The conjugation of whole antibodies to the liposome surface can induce complement activation and decrease their blood circulation since the Fc fraction of immunoglobulins is recognized by macrophages [45, 131]. Thus conjugation of Fab' fragments instead of the whole antibody was proposed. While doxorubicin-loaded PEGylated immunoliposomes harboring Fab' fragments of an anti-CD19 antibody had similar blood circulation and MPS accumulation than untargeted liposomes, immunoliposomes harboring the anti-CD19 IgG showed faster blood clearance and a threefold accumulation in liver and spleen over untargeted or Fab' liposomes [101]. Fab' immunoliposomes also resulted in superior therapeutic efficacy over untargeted or anti-CD19 antibody-decorated immunoliposomes [101]. Analogous with their results, the blood circulation of pH-sensitive 1-D-arabinofuranosylcytosine-loaded liposomes harboring Fab' fragments against CD33 was superior to those decorated with the whole monoclonal antibody [121].

#### 2.2.2. Protein-Targeted Liposomes

Qi et al. described a novel antineoplastic liposomal agent, liposomal saposin C [132]. Development of this agent is based on the observation that patients suffering from lysosomal storage diseases frequently have saposin C deficiencies leading to accumulation of toxic glycosylceramide sphingolipids [133] and that saposin C inserts into negatively charged membranes at acidic pH [134]. They prepared a saposin C-DOPS conjugate which assembled as 190 nm liposomes under sonication at acidic pH. Tumor targeting is based on activation of membrane fusion domains of saposin C at the acidic pH in tumors leading to its internalization and glycosylceramide-induced apoptosis. Intravenous injection into neuroblastoma xenograft- bearing mice led to apoptosis induction in tumors and tumor growth inhibition without systemic toxicity. BAFF (B cell activating factor) is a cytokine whose receptor is overexpressed in B-cell lymphomas, conjugation of a BAFF mutant to vincristine-loaded PEGylated liposomes increased the survival of lymphoma-bearing mice over untargeted vincristine-loaded liposomes or free drug [35]. Cancer cells overexpress transferrin receptors [135] making the glycoprotein, transferrin or antibodies to transferrin receptor, suitable ligands for tumor targeting [136]. Addition of transferrin to the surface of PEGylated oxaliplatin-loaded liposomes increased tumor accumulation over free oxaliplatin or untargeted liposomes leading to the highest tumor growth inhibition against C26 colon carcinoma-bearing mice [36]. In parallel to these studies, conjugation of transferrin to doxorubicin-loaded liposomes resulted in higher doxorubicin delivery to tumors and tumor growth inhibition over untargeted doxorubicin-loaded liposomes [103].

#### 2.2.3. Peptide-Targeted Liposomes

More and more tumor-specific ligands are being identified by combinatorial screening of bacteriophage-borne peptide libraries, phage display biopanning. This is a strategy whereby the recombinant virions able to bind cancer cells *in vitro* or tumors *in vivo* are purified before identification of the peptide and its use for targeted drug delivery, allowing identification of peptides specific for cancer cells, tumor vasculature or both (reviewed in [137]). 

We previously described the selective exposure of nucleohistones by cancer cells effective cancer therapy of antinuclear-targeted doxorubicin-loaded liposomes [32]. In good agreement with these studies, Wang et al. reported tumor targeting of doxorubicin-loaded liposomes harboring the histone H1-specific peptide ApoPep-1 [138]. This peptide is selectively presented at the surface of tumor cells due to spontaneous apoptosis in avascular tumors. ApoPep-1 conjugation to doxorubicin-loaded liposomes led to superior doxorubicin distribution in lung xenografts and better tumor growth inhibition over untargeted liposomes. Somatostatin receptors, particularly somatostatin receptor type 2, are overexpressed by cancer cells and endothelial cells of the tumor vasculature [139]. Coupling of the somatostatin receptor type 2 agonist to irinotecan-loaded liposomes improved their antitumor activity in a medullary thyroid carcinoma model [105]. Its coupling to PEGylated doxorubicin-loaded liposomes led to superior doxorubicin accumulation in tumors and enhanced anticancer efficacy against small cell lung cancer tumors compared to untargeted liposomes [106]. 

Han and coworkers selected a peptide (HVGGSSV) by phage display which selectively bound to the tumor vasculature of tumors that were regressing after radiotherapy, while no binding was detected before irradiation or in areas of tumor necrosis factor alpha-induced inflammation in mice [140]. They proposed the peptide that recognized a protein displayed only on tumor endothelial cells that were responding to therapy. Interestingly, they conjugated this peptide to the surface of doxorubicin-loaded liposomes for “radiation-guided tumor-targeted drug delivery” [141]. Higher tumor accumulation of doxorubicin was achieved with targeted liposomes after irradiation over untargeted doxorubicin-loaded liposomes with or without irradiation and resulted in higher therapeutic efficacy in both Lewis lung carcinoma and non-small cell lung carcinoma (HL460) tumors. Identification of a non-small cell lung cancer-specific peptide also identified by phage display to doxorubicin or vinorelbine-loaded PEGylated liposomes enhanced drug distribution to tumors and resulted in increased therapeutic efficacy over untargeted drug-loaded liposomes [38]. Another group reported higher therapeutic efficacy against lung cancer xenografts of PEGylated doxorubicin-loaded liposomes conjugated with a large-cell cancer-specific peptide over untargeted doxorubicin-loaded liposomes [142]. 

Breast cancer-specific peptide/phage fusion coat protein pVIII chimeras have been used for tumor-targeted drug delivery [143, 144]. Membranophilic major phage coat protein pVIII fused with a targeting peptide identified by phage display spontaneously inserts into liposomes. The insertion of a breast cancer-specific phage fusion protein into doxorubicin-loaded liposomes (Doxil) led to an increased binding to breast tumor cells and enhanced cytotoxicity over untargeted Doxil liposomes *in vitro* [143, 144]. This is noteworthy, since no chemical conjugation step is involved, this method allows fast and selective identification of tumor ligands.

PEGylated paclitaxel-loaded liposomes harboring a synthetic luteinizing hormone-releasing hormone (LHRH) peptide designed to interact with the LHRH receptors that are overabundant in the membrane of cancer cells [145] showed increased tumor accumulation and therapeutic efficacy over untargeted paclitaxel-loaded liposomes [107]. Matrix metalloproteinases (MMPs) are overabundant in tumor tissues where they act in angiogenesis, matrix degradation, and metastasis [146]. Moreover, MMP-2/*α*_V_ *β*_3_ integrin complexes and MMP-9 are present at the surface of angiogenic blood vessels and cancer cells, respectively and their targeting by inhibitory peptides showed antitumor effects [147, 148]. MMP-targeting of Caelyx doxorubicin-loaded liposomes by insertion of a DSPE-PEG3400-CTT2 conjugate, the CTT2 peptide binding to MMP 2 and 9, led to increased doxorubicin accumulation in tumors and extended the survival of ovarian carcinoma xenograft-bearing mice over unmodified Caelyx liposomes [40].

#### 2.2.4. Small Molecule-Mediated Tumor Targeting

Aberrant tumor growth is correlated with a greater demand for nutrients relative to healthy organs and has been exploited for tumor targeting. To sustain their rapid growth, tumor cells overexpress folate receptor to capture the folate required for DNA synthesis [149]. The overexpression of folate receptor in cancers of several histology relative to normal tissues, the low cost of folic acid (FA), and the vast library of conjugation reactions available make it one of the most used ligands for tumor-targeted drug delivery and tumor imaging (reviewed in [150]). Inclusion of a FA-PEG-DSPE conjugate into irinotecan-loaded liposomes enhanced drug concentration in tumors after intravenous injection over untargeted liposomes or free irinotecan resulting in the highest anticancer activity without detected side toxicity [41]. Similarly, folate-targeting of doxorubicin-loaded liposomes increased the survival of tumor bearing mice by 50% over untargeted liposomes [111]. Lee et al. used tetraiodothyroacetic acid, a competitive inhibitor of thyroid hormone binding to the endothelial cell integrin *α*_*V*_ *β*_3_, as a new ligand for tumor-targeted drug delivery. This ligand increased liposomal accumulation in tumors after intravenous injection and enhanced anticancer activity of the encapsulated anticancer drug edelfosine [151]. 

Estrogen receptors are often overexpressed in breast and ovarian cancers and conjugation of the ovarian estrogenic hormone estrone to doxorubicin-loaded liposomes resulted in a dramatic increase in doxorubicin accumulation in breast tumors after intravenous injection over free drug or untargeted PEGylated doxorubicin-loaded liposomes (24.3 and 6.0-fold, resp.) resulting in the highest therapeutic activity [42, 112]. Similarly, conjugation of a luteinizing hormone-releasing hormone (LHRH) analog to the surface of docetaxel-loaded liposomes increased docetaxel accumulation in ovarian xenografts by 2.86-fold over untargeted docetaxel-loaded liposomes with decreased liver and spleen capture though binding to the LHRH receptors highly overexpressed in ovarian cancer [152]. The basic fibroblast growth factor (bFGF) receptor is also overexpressed in several cancers [153]. Electrostatic coating of cationic liposomes encapsulating doxorubicin or paclitaxel with a negatively charged bFGF-derived peptide resulted in increased survival of melanoma or prostate tumor-bearing mice over untargeted liposomal formulations, respectively [154]. The use of chondroitin sulfate which binds CD44 overexpressed by tumor cells has recently been introduced [43]. Coupling of chondroitin sulfate to the surface of etoposide-loaded liposomes increased etoposide accumulation in breast cancer xenografts after intravenous injection 40-fold compared to free drug and by 8-fold compared to untargeted liposomes. Presentation of lactose at the surface of doxorubicin-loaded PEGylated liposomes using a lactose-DOPE conjugate to target the asialoglycoprotein receptors overexpressed in hepatocellular carcinomas increased doxorubicin accumulation in tumors and resulted in tumor growth inhibition over untargeted doxorubicin-loaded liposomes [116]. 

Tan and coworkers introduced ternary nucleic acid complexes, Liposome Polycation DNA (LPD) where nucleic acids are complexed by protamine before interaction with cationic liposomes to form a core nucleic acid complex surrounded by two lipid bilayers [155]. Sigma receptors are ion channel regulators overexpressed in several cancer types [156] Conjugation of the small molecular weight sigma receptor ligand anisamide, [157] to the distal end of PEG2000-DSPE allowed 70–80% luciferase silencing in an experimental lung metastasis model [113]. Moreover, parenteral injection of anisamide-armed LPD prepared with a combination of siRNA against the inhibitor of p53, MDM2 (Murine Double Minute 2), against the Cmyc oncogene and the other against the angiogenesis regulator, VEGF (Vascular Endothelial Growth Factor) were localized in tumors and allowed a 70–80% decrease in tumor load [68]. However, while the common sigma receptor agonist haloperidol and anisamide recognize sigma receptor type 1 and 2, only sigma receptor type 2 overexpression has been reported to be a prognostic indicator [158]. The latter has low expression in healthy tissues, suggesting a higher therapeutic index of sigma receptor 2 targeted therapies [158]. Indeed, binding of the sigma 2 receptor agonist SV119 to its receptor induced cell death *in vivo* in a pancreatic cancer model, and conjugation of SV119 to the surface of liposomes increased their uptake *in vitro* in cell lines including lung, breast, and prostate cancer carcinoma whereas no increased uptake in normal cells was reported [158].

## 3. Biological Targets

### 3.1. Brain Tumor Targeting

Brain tumors are a major concern for both primary brain and brain metastases from primary lung, melanoma, breast, and kidney cancers [159]. Therapy against brain cancers is challenging since the brain is largely isolated from the rest of the body by the blood brain barrier (BBB), a dense barrier of endothelial cells, pericytes, astrocytes, and extracellular matrix which limits molecular transport into the brain [160]. Several strategies to overcome this barrier have been proposed for the treatment of brain tumors, either by targeted delivery of drug-loaded liposomes to the brain or by remote-controlled drug release within the brain.

Overexpression of IL-13 receptors has been reported in human gliomas [161], and conjugation of IL-13 to doxorubicin-loaded liposomes allowed a 5-fold reduction in tumor volume and extended survival of intracranial glioma tumor-bearing mice over untargeted doxorubicin-loaded liposomes [104]. In the same vein, the conjugation of IL-13 to PEGylated doxorubicin-loaded liposomes for astrocytoma targeting dramatically improved brain delivery of doxorubicin compared to untargeted liposomes and resulted in increased survival of intracranial U87 glioma-bearing mice after intraperitoneal administration [104]. To reinforce brain drug delivery, Du et al. armed PEGylated topotecan-loaded liposomes with both wheat germ agglutinin for brain capillary targeting and tamoxifen to decrease drug efflux [162]. These dual-targeted liposomes crossed a model BBB* in vitro* and increased the survival of brain tumor bearing-rats over free topotecan or untargeted topotecan-loaded liposomes [162]. The need for dual-targeting for effective BBB crossing *in vivo* is also exemplified in a study by Ying et al. [163]. They took advantage of the expression of glucose transporter 1 and transferrin receptor by endothelial cells of the BBB for intracranial glioma therapy using mannose and transferrin dual-targeted daunorubicin-loaded liposomes. Dual-targeting led to superior tumor growth inhibition and increased life span over untargeted or single-targeted daunorubicin-loaded liposomes. 

Gong et al. used thermosensitive doxorubicin-loaded PEGylated liposomes capable of releasing 90% of drug after 30 min at 42°C compared to less than 3% for unsensitive liposomes [164]. They reported improved doxorubicin delivery to the brain after intravenous injection (3.4-fold over nonsensitive liposomes) and increased survival of C6 glioma-bearing mice when heads of mice were heated in a water bath to 42°C after injection [164]. Another physically controlled content release strategy has been described by the group of Yang using focused ultrasounds for reversible disruption of the BBB as evidenced by higher brain accumulation of Evan's blue or gadolinium in ultrasound-treated animals over untreated ones [165]. Administration of brain tumor-targeted doxorubicin-loaded liposomes followed by ultrasound-mediated BBB disruption allowed higher levels of intracranial liposomes and doxorubicin accumulation over untargeted liposomes in an intracranial glioblastoma model [166]. 

### 3.2. Vasculature Targeting

The “angiogenic switch,” when tumors establish their own blood supply by extensive neo-angiogenesis, is critical for the progression of tumors from a dormant avascular nodule to an invasive carcinoma [167, 168]. This dependence on blood supply for tumor growth and the correlation between vascular permeability and accumulation of liposomal drug and therapeutic efficacy [169–171] supports research on liposomal tumor vasculature-targeting for cancer therapy (reviewed in [172]). After intravenous injection in mice, PEGylated liposomes were shown to accumulate in the perivascular space with limited tumor penetration [94, 173, 174]. Moreover, when the tumor accumulation and therapeutic efficacy of PEGylated liposomal oxaliplatin were compared in animals bearing C26 colon carcinoma, Lewis lung carcinoma and B16BL6 melanoma, a correlation among tumor blood vessel permeability, tumor drug accumulation and the resulting therapeutic efficacy have been reported [171]. *In vitro* results were not predictive of *in vivo* activity: the least tumor accumulation and tumor growth were detected in B16BL6 tumors, whereas this cell line was the most sensitive to liposomal oxaliplatin *in vitro*, [171]. Of note, the lower tumor vessel permeability of melanoma xenografts compared to colon or lung carcinoma is clinically relevant. When the microvessel density of biopsies from cancer patients was determined, melanoma was also the least vascularized (~35 vessels/field) compared to colon (~70) or lung tumors (~127), stressing the point that extravasation of agents from the tumor vasculature is a major barrier for liposomal drug delivery [175]. 

Targeting of selectin on endothelial cells with P-selectin glycoprotein ligand 1 allowed a 3-fold higher luciferin delivery to B16F10 tumors after intravenous injection over untargeted liposomes [176]. The *α*_*V*_ *β*_3_ integrin is overexpressed by endothelial cells in the tumor vasculature [177]. The tripeptide Arg-Gly-Asp (RGD) and the cyclic RGD (Arg-Gly-Asp-D-Phe-Lys) are *α*_*V*_ *β*_3_ ligands used for tumor-targeted drug delivery [108]. RGD-targeted paclitaxel or doxorubicin-loaded PEGylated liposomes showed superior therapeutic activity over free drug or untargeted liposomes [109, 110]. Antitumor activity of RGD-targeted liposomes is consistent with tumor microvessel destruction after injection of RGD-targeted paclitaxel-loaded liposomes reported by another group [178]. Functionalization of doxorubicin-loaded liposomes with a peptide targeted to bombesin receptors overexpressed in cancers improved therapeutic efficacy over untargeted liposomes [179]. *α*_5_ *β*_1_ is another integrin overexpressed in cancer in which the fibronectin-derived peptide antagonist ATN-161 showed antineoplastic and antimetastatic properties [180]. Coupling of ATN-161 to doxorubicin-loaded PEGylated liposomes increased their therapeutic activity in a melanoma model [181]. Doxorubicin-loaded PEGylated liposomes were functionalized with a NGR peptide at the distal end of PEG to target a CD13 isoform overexpressed in the tumor neovasculature [182–184]. In the study by Pastorino et al., vasculature-targeted Caelyx showed superior apoptosis induction in tumor xenografts and decreased blood vessel density leading to increased survival of mice bearing lung, ovarian, or neuroblastoma xenografts compared to untargeted Caelyx [182]. 

To further improve the destruction of blood vessel support of tumors, Takara and coworkers recently developed a dual-ligand approach for antiangiogenic therapy using liposomes targeted to CD13 (NGR-PEG2000-DSPE) functionalized with the stearylated cell penetrating peptide tetra-arginine at the liposome surface [183]. They first compared endothelial cell association *in vivo* in tumor-bearing mice after intravenous injection of PEGylated doxorubicin-loaded liposomes measuring either 100 nm (small liposomes) or 300 nm (large liposomes). Since a superior association with tumor blood vessels and lower extravasation was observed with large liposomes over small ones, they used the former for ligand conjugation. Dual-ligand labeled liposomes accumulated ~3-fold more in tumors than unmodified or single ligand-modified liposomes, revealing synergy of the two ligands. Consistent with the tumor accumulation and blood vessel association results, only the dual-ligand doxorubicin-loaded liposomes allowed protection against tumor growth and induced tumor blood vessel destruction that revealed a synergy of endothelial cell targeting and enhanced uptake for antiangiogenic therapy.

Cationic liposomes selectively bound to endothelial cells *in vivo* with superior internalization over anionic or neutral liposomes due to the enrichment of tumor endothelial cell membranes with negatively charged lipids and heparan sulfate proteoglycan [172, 185, 186]. Superior accumulation of oxaliplatin in lung tumors was obtained after intravenous injection of PEG-coated cationic drug-loaded liposomes over neutral liposomes [187]. The same group used cationic liposomes for delivery of siRNA against the neoangiogenesis regulator, Argonaute 2 (Ago2) which resulted in Ago silencing in tumors together with apoptosis of tumor blood vessels and decreased tumor growth while no therapeutic effect was observed with cationic lipoplexes prepared with an irrelevant siRNA [188, 189]. In support of the effect of the negative charge of angiogenic vessels, paclitaxel-loaded cationic liposomes (EndoTAG-1) induced endothelial cell apoptosis *in vivo,* retarded melanoma and pancreatic carcinoma tumor growth, and decreased the number of melanoma lung metastases *in vivo* [190–192]. Recently, targeting of tumor vasculature by an aptamer directed against the tumor vasculature marker E-selectin has been reported [193]. E-selectin aptamer conjugated liposomes accumulated in the tumor vasculature of breast cancer xenografts after intravenous injection, whereas no untargeted liposomes were detected in tumors, supporting use of this selective approach for vasculature-targeted drug delivery. The vasculature-targeting group used may be relevant only to a particular histology. Indeed, while the p15-RGR peptide which recognizes platelet-derived growth factor receptor *β* expressed by pericytes of the tumor vasculature identified by phage display against pancreatic cancer increased delivery of liposomes to pancreatic tumors *in vivo*, it did not direct liposomes to tumors in a melanoma model [194, 195]. In the same study, liposomes harboring p46-RGD *α*_V_-integrin-binding peptide targeting tumor endothelial cells allowed a significant tumor accumulation over controls with higher therapeutic efficacy [195]. Chang et al. also used phage display to identify neovasculature peptides which when conjugated to doxorubicin-loaded liposomes increased doxorubicin delivery to tumors and therapeutic efficacy over untargeted PEGylated doxorubicin-loaded liposomes [196].

Pericytes are a critical conjunctive component of vasculature; aminopeptidase A (APA) has been identified as a marker of pericytes from orthotopic primary and metastatic (ovary) neuroblastoma in mice [197]. Coupling of a peptide ligand of APA to doxorubicin-loaded liposomes increased doxorubicin accumulation in neuroblastoma tumors over untargeted doxorubicin with better therapeutic activity demonstrating that pericytes are another critical target within the vasculature [198]. Moreover, coadministration of APA-targeted doxorubicin-loaded liposomes and aminopeptidase N (APN, a marker of tumor endothelial cells) targeted doxorubicin-loaded liposomes led to superior doxorubicin accumulation in tumors over either targeted formulation alone [198]. The destruction of perivascular and endothelial cells in tumors resulted in a significant increase in survival of neuroblastoma-bearing mice over either endothelial cell-targeted or pericyte-targeted liposomes alone [198]. 

Tumor lymphatics are also a therapeutic target since they support lymph node metastasis [199]. Indeed, lymph node invasion is frequent in melanoma and is an indicator of poor prognosis [200]. Laakkonen and coworkers identified a tumor lymphatics-binding peptide (LyP-1) which, after intravenous injection in breast carcinoma-bearing mice, was shown to accumulate in hypoxic areas of primary tumors, cofllocalize with lymphatic markers in primary tumors and lymph node metastases leading to tumor growth reduction and a decreased number of lymphatic vessels [201, 202]. Interestingly, presentation of this peptide on doxorubicin-loaded liposomes increased tumor accumulation and therapeutic efficacy over untargeted liposomes and decreased lymph node metastasis rate and growth [201, 203–205].

A combination of targeting ligands may be needed for effective antiangiogenic therapy. Murase et al. demonstrated synergy in association with endothelial cells *in vitro* by liposomes modified with two angiogenic vessel-targeted peptides (APRPG and GNGRG) identified by phage display and revealed the more intense association with tumor blood vessels *in vivo* of dual-targeted liposomes over single-modified liposomes [206]. Similarly, Meng et al. demonstrated synergy in tumor growth inhibition of non-small cell lung cancer of PEGylated paclitaxel-loaded liposomes targeted to tumor vasculature by both RGD and a neuropilin 1-specific peptide over untargeted or single-targeted liposomes [207]. These results are in accordance with the increased detection of neoangiogenic blood vessels in surgical specimens from cancer patients when using two neovasculature-specific peptides simultaneously compared to individually used [196].

### 3.3. Targeting and Inhibition of Metastasis

Metastasis is the ultimate stage of clinical cancer and is the stage with the least survival. Treatment of metastasis is challenging because micrometastatic foci are hard to detect and more aggressive than the primary tumors [208]. Elimination of metastases is thus of utmost importance to prevent cancer recurrence after chemotherapy or surgical removal of the primary tumor. Platelets have been proposed as shuttles for tumor cell metastasis by formation of platelets-tumor cell aggregates [209, 210]. This is consistent with the elevated platelet counts in patients with advanced cancer [210]. Therefore, Wenzel et al. used PEGylated liposomes to codeliver the haemostatic inhibitor dipyridamole (DIP) and the cytotoxic drug perifosine (OPP) to inhibit platelet-tumor cell aggregate formation and kill tumor cells, respectively [211]. OPP/DIP coloaded liposomes inhibited aggregation of platelets, decreased formation of platelet-tumor cell aggregates *in vitro* and decreased the number of experimental lung metastases when intravenously injected 6 h before parenteral injection of tumor cells. The metastasis-specific peptide TMPT1 [212] recognizes highly metastatic primary tumors and metastases of prostate, breast, and lung cancers relative to their nonmetastatic counterparts. Conjugation of this peptide to doxorubicin-loaded liposomes led to deeper tumor penetration and greater induction of apoptosis with superior tumor growth inhibition against highly metastatic breast cancer xenografts [39]. PAR-1 (Protease Activated Receptor 1), a thrombin receptor, is a major regulator of metastasis in melanoma through its roles in matrix degradation and angiogenesis [213]. Villares et al. reported for the first time a dramatic antimelanoma therapeutic activity after systemic delivery of PAR-1 siRNA-loaded neutral DOPC liposomes with tumor weight reduction and a decrease in experimental lung metastatic colonies [214]. This was achieved via downregulation of promoters of angiogenesis (VEGF and IL-8) and invasion (MMP-2) together with decreased tumor blood vessel density (decreased CD31 staining). 

### 3.4. Immune Cell Targeting

For therapeutic vaccination against cancer, patient's immune cells are stimulated by tumor cell antigens. Since the development of effective adaptive immune responses by CD4^+^ T cells or CD8^+^ T cells with cytotoxic activity (Cytotoxic T Lymphocytes, CTL) requires their activation by dendritic cells (DCs) that present tumor antigen peptides [215], their targeting is of therapeutic relevance [215–217]. Altin's group used a chelator lipid [Nickel/3(nitrilotriacetic acid)-ditetradecylamine], (Ni-NTA_3_-DTDA) for functionalization of liposomes with histidine-tagged peptides though polyhistidine binding to nitrilotriacetic acid in the presence of nickel [218, 219]. For antigen delivery, Ni-NTA_3_-DTDA functionalized liposomes were prepared by preinsertion before conjugation with histidine-tagged peptides derived from ICAM4 (Intercellular Cell Adhesion Molecule 4), a ligand of the murine dendritic cell (DC) integrin CD11c/CD18 [220]. Ovalbumin-loaded PEGylated liposomes decorated with DC-targeting peptides distributed to splenic DC *in vivo*, induced an adaptive immune response against, ovalbumin and exhibited dramatic therapeutic activity against established B16-OVA melanoma tumors with complete tumor regression in 80% of treated mice [218]. 

In other studies Altin's group reported on DC-targeted gene delivery *in vivo* and potent antitumor effects in the B16-OVA melanoma model after liposome functionalization with histidylated flagellin, the major constituent of the bacterial flagella, recognized by the Toll Like Receptor 5 that leads to their activation [221, 222]. LPR (Lipid-Polymer-RNA) mannosylated and histidylated lipopolyplexes loaded with MART1 (Melanoma Antigen Recognized by T cells 1) mRNA delayed the progression of B16F10 melanoma more effectively than untargeted LPR [223]. This study also illustrated the importance of cytosolic delivery of nucleic acids for *in vivo* transfection of DC. The authors used a ternary formulation of mRNA or pDNA coding for the reporter gene EGFP (Enhanced Green Fluorescent Protein) complexed with PEGylated histidylated poly-L-Lysine and imidazole-rich liposomes, both of which promote endosomal escape [224, 225]. While no *in vivo* transfection of splenic DC was observed with pDNA, 12% were transfected with mRNA mannosylated LPR and 3% with untargeted LPR demonstrating that nuclear delivery is a limiting step for DC transfection. Liposomes targeted to dendritic cells by mannosylated ligands have recently been used as a platform for effective cancer immunotherapy [114]. The liposomes used harbored mannosylated ligands at their surface for targeting of antigen presenting cells with a cytotoxic T lymphocyte peptide of the renal carcinoma antigen ErbB2 for induction of an adaptive immune response, Toll Like Receptors (TLRs) agonists as adjuvants and a T helper lymphocyte epitope peptide for improved immune activation. Of note, the authors developed new functionalized lipid anchors devoid of adjuvant activity for their study: dipalmitoylglycerol maleimide and dipalmitoylglycerol bromoacetate. These liposomes induced an adaptive immune response against the ErbB2 antigen with high therapeutic activity. Targeting of intraperitoneal macrophages by ovalbumin-loaded liposomes armed with dipalmitoylphosphatidylethanolamine conjugated mannotriose increased antigen-specific cell lysis induction by splenocytes over untargeted liposomes resulting in therapeutic efficacy both as a preventive and therapeutic cancer vaccine [115]. In addition to carrying tumor antigens, liposomal vaccines are armed with immunostimulatory lipids, usually derived from microorganisms, recognized by pathogen recognition receptors leading to immunostimulation (reviewed in [226]). Zhong et al. compared the antimetastatic efficacy of a basic Fibroblast Growth Factor (bFGF) vaccine in a mouse melanoma model when administered as a Freund's adjuvant mixture, in cationic liposomes, or cationic liposomes containing 0.25% of monophosphoryl lipid A as adjuvant [227]. They reported higher anti-bFGF IgG titers and higher pulmonary metastasis inhibition in mice treated with monophosphoryl lipid A bFGF-loaded liposomes over cationic liposomes or a bFGF/Freund's adjuvant mixture without the toxicity associated with administration of free adjuvants.

Selective depletion of tumor supporting cells represents another approach to cell-specific cancer therapy [228]. The tumor environment is enriched in tumor supporting cells among the tumor-associated macrophages that constitute a predominant inflammatory population involved both in resistance to therapy and metastasis [228]. Dichloromethylenediphosphonate (DMDP) liposomes induced macrophage depletion after intravenous injection in mice [229]. Intradermal injection of DMDP liposomes into the tissues surrounding melanoma or squamous cell carcinoma tumors led to a decrease in tumor-associated macrophages content and tumor rejection [230].

Ligand density was shown to influence both drug retention and target recognition. Zhang et al. demonstrated increase in liposome uptake *in vitro* as the ligand density was increased from 0% to 1, 3, and 5% demonstrating enhanced ligand recognition [231]. However, increase of *in vitro* drug release as a function of DSPE-PEG-RGD ligand moiety has been reported by others [232]. Moreover, Saul et al. evidenced increase of nonspecific uptake *in vitro* with ligand density [233]. Consistent with their results, lower tumor accumulation of NGR (Asparagine-Glycine-Arginine) vasculature targeted liposomes has been evidenced *in vivo* with liposomes harboring 2.56% mole NGR-PEG-DSPE than 0.64% mole NGR-PEG-DSPE [234]. Altogether, these data suggest the use of the lowest targeting ligand density allowing target binding for effective anticancer therapy. 

## 4. Liposomes for Combination Therapy

The prevalence of drug resistance in cancer patients, both prior to treatment and *de novo* [235, 236], fueled the application of drug combinations to treat cancer as an alternative to increased doses of chemotherapeutics associated with life threatening sideeffects [237–239].

Codelivery was well illustrated in a study by Chen et al. [240]. Using LPH-NP (liposome-polycation-hyaluronic acid) nanoparticles targeted by postinsertion of DSPE-PEG-GC4 (scFv selected by phage display against ovarian tumors [241]), they codelivered 3 different siRNA and one miRNA and obtained a 80% decrease in tumor load after treatment. They simultaneously targeted proliferation pathways with Cmyc siRNA and miR34a miRNA [242, 243], apoptosis with MDM2 siRNA [244], and angiogenesis using VEGF siRNA [245]. Liposomal codelivery of siRNA against the apoptosis regulator Mcl-1 (Myeloid cell leukemia sequence 1) and of the MEK (Mitogen-activated Extracellular Kinase) and apoptosis resistance inhibitor PD0325901 enhanced tumor growth inhibition compared to each treatment alone [246]. The same group also developed trilysinoyl oleyamide (trilysine peptide linked to oleyamine by a peptide bond) based PEGylated liposomes for codelivery of Mcl-1 siRNA and the histone deacytylase inhibitor suberoylanilide hydroxamic acid (SAHA) [247]. Intravenous administration increased the tumor growth delay compared to liposomes with SAHA and an irrelevant siRNA. Likewise, Xiao and coworkers used targeted liposomes to codeliver doxorubicin and DNA encoding a dominant mutant of survivin [248]. Liposomes were targeted by a truncated basic fibroblast growth factor (tbFGF) peptide recognizing the bFGF receptor upregulated in lung cancers and contained doxorubicin and pDNA encoding for a dominant negative mutant of survivin to counter survivin-mediated apoptosis resistance [249]. Their codelivery produced a higher therapeutic efficacy against Lewis lung carcinoma tumors than liposomes with either agent alone. 

A further step in combination of an antineoplastic agent with modulation of drug resistance was achieved recently by Minko and coworkers [250] by formulation of peptide-targeted liposomes containing doxorubicin or cisplatin together with oligonucleotides against the two main drug resistance mechanisms Bcl-2 and MDR1. The efficacy of this “combined targeted chemo and gene therapy” system was evaluated in xenografts established from human ovarian malignant ascites. While inclusion of either Bcl-2 or MDR1 antisense oligonucleotides in cisplatin or doxorubicin-loaded targeted liposomes decreased primary tumor volume and intraperitoneal metastases load, further inhibition of tumor growth inhibition was obtained with targeted liposomes containing doxorubicin or cisplatin, Bcl-2 and MDR1 antisense oligonucleotides together with complete prevention of the development of detectable intraperitoneal metastases or ascites. Interestingly, Minko et al. proposed this system as a platform for personalized cancer therapy with liposomal formulations containing antisense oligonucleotides targeting individually relevant resistance mechanism. Sawant et al. coloaded PEGylated liposomes with a palmitoyl-ascorbate conjugate and paclitaxel [251]. The therapeutic benefit of the coloading against 4T1 mammary carcinoma was evident at 10 mg/kg compared to palmitoyl-ascorbate or paclitaxel-loaded liposomes. Atu027 (Silence Therapeutics, London, UK) is a liposomal formulation of siRNA against protein kinase N3, a downstream effector of the mitogenic PI3 K/PTEN pathway involved in prostate cancer metastasis [252, 253]. This formulation was composed of 2′-*O*-methyl-stabilized siRNA encapsulated in cationic liposomes (50 mol% cationic lipid -L-arginyl-2,3-L-diaminopropionic acid-N-palmitoyl-N-oleyl-amide trihydrochloride (AtuFECT01), 49 mol% co-lipid 1,2-diphytanoyl-sn-glycero-3-phosphoethanolamine (DPhyPE), and 1 mol% DSPE-PEG2000) [253]. This formulation showed very promising results in phase I clinical trial with tumor regressions in neuroendocrine and breast cancer patients [254]. 

Dai et al. combined targeted delivery with antineoplastic and antiangiogenic agent delivery in PEGylated liposomes [255]. Coloading of the antiangiogenic agent combretastin A-4 in the lipid bilayer and the anticancer drug doxorubicin in the aqueous core of PEGylated liposomes resulted in increased therapeutic activity. Hu et al. also combined liposomal delivery of the antineoplastic and antiangiogenic agent, honokiol with irradiation for maximal therapeutic efficacy [256]. They hypothesized that this protocol would combine the destruction of tumor cells by irradiation with inhibition of irradiation-induced neoangiogenesis by honokiol [257]. The combination of PEGylated honokiol-loaded and radiotherapy showed increased survival of Lewis lung carcinoma-bearing mice compared to radiotherapy or honokiol liposomes alone, resulting in decreased angiogenesis *in vivo*. Maitani et al. also combined an antineoplastic drug (irinotecan) and an antiangiogenic agent (sunitinib) [258]. The drug combination had more therapeutic efficacy against pheochromocytoma neuroendocrine tumors *in vivo* when they were administered as sunitinib liposomes plus irinotecan liposomes or as coloaded liposomes than the combination of the free drugs, with higher drug accumulation as liposomes than as free drug. In a similar fashion, folate-targeted doxorubicin-loaded liposomes coloaded with a bifunctional peptide capable of vascular disruption and antitumor activity were more effective against KB human nasopharyngeal carcinoma *in vivo* than untargeted coloaded liposomes than either monotherapy [259]. RGD-targeted liposomes coloaded with doxorubicin and the vascular disrupting drug combrestatin A-4 increased tumor regression of B16F10 melanoma compared to untargeted coloaded liposomes or targeted liposomes with either drug [260].

Zucker and coworkers have optimized the simultaneous loading of vincristine and topotecan into PEGylated liposomes (LipoViTo liposomes) and provided the reader with the methods needed to characterize a liposomal drug combination [261]. Use of LipoViTo increased 100-fold the drug distribution to tumors compared to free drug and led to superior therapeutic efficacy over a free drug combination or liposomes with a single drug. PEGylated liposomes containing both vincristine and quercetin allowed reduced blood clearance of both drugs in mice, increased the therapeutic activity over a combination of free drugs and decreased side-toxicity [262]. 

Celator Pharmaceuticals Inc. (Princeton, NJ) developed a liposomal formulation of cytarabine: daunorubicin (CPX-351, 5 : 1 molar ratio) [24, 263, 264]. These PEGylated liposomes coloaded with the weak acid drug, 5-fluoroorotic acid (FOA) and the amphiphatic drug, irinotecan (CPT-11) at a 5 : 1 ratio revealed a synergy between the two drugs with higher therapeutic efficacy than the free drug cocktails in animal models [264, 265]. To encapsulate both drugs, they first prepared liposomes before active loading of CPT-11 by a pH gradient method, with the protonated CPT-11 retained in liposomes after complex formation with FOA. Mice treated with coloaded liposomes had increased survival compared to the combination with separate liposomes. However, the therapeutic efficacy was lower than with liposomes loaded with FOA only, probably because the FOA content had to be lowered for CPT-11 coloading, further demonstrating the difficulty of reproducing a synergy with liposomes relative to free drugs. When tested in phase I trial with acute leukemia patients, the 5 : 1 ratio was maintained in plasma for 24 h, and CPX-351 induced complete responses in 9 out of 43 patients [24]. The same group developed irinotecan: floxuridine liposomes (CPX-1, 1 : 1 molar ratio). In phase I clinical trial they demonstrated that the drug ratio was maintained in plasma up to 12 h after infusion and showed positive clinical responses in patients with colorectal cancer [25]. It is noteworthy that the high therapeutic efficacy of liposomes encapsulating two anticancer drugs was always correlated with the maintenance of their synergistic molar ratio in plasma, in animal models [266] as well as in cancer patients [24, 25, 264] indicating optimization of drug loading and liposomal stability as primary concerns for effective combination therapy. Ko et al. codelivered the proapoptotic peptide D-(KLAKKLAK)_2 _and the Bcl-2 antisense oligodeoxynucleotide G3139 [267]. The authors took the advantage of the electrostatic properties of these therapeutic molecules to codeliver them by formation of a negatively charged complex between the peptide and G3139 before mixing with positively charged liposomes. Intratumoral injection of coloaded liposomes led to an enhanced tumor growth suppression.

Finally, the combined liposomal delivery of magnetic fluid hyperthermia and photodynamic therapy using magnetic fluid and zinc phthalocyanine as the photosensitizer demonstrated superior toxicity *in vitro* of combined light and magnetic stimuli over their separate applications suggesting a new treatment modality for enhanced tumor therapy [268].

## 5. Tumor Stimuli-Triggered PEG Release

The addition of PEG to the liposome surface was reported to decrease the interaction of the ligand-targeted liposomes with their ligand, either when small molecules were conjugated to the liposome surface [269] or with antibody-targeted liposomes [48, 118] by steric hindrance of the surface ligand. Moreover, PEGylation decreases targeted liposomal accumulation and drug release [270]. Finally, for gene delivery, PEGylation has been shown to decrease intracellular trafficking of DNA [271]. These drawbacks and the extensive research in PEGylation chemistry (recently reviewed in [272, 273]) have led to the preparation of new multifunctional carriers where PEG release is promoted at the tumor's vicinity after a stimulus either by physiological stimuli (pH, altered redox potential, sensitivity to an enzyme overabundant in the tumor microenvironment) or by physical external stimuli (light, heat, and ultrasound) [8, 274] (Figure 2).

### 5.1. pH-Sensitive PEG Release

While normal tissues and blood have a physiological pH near 7.4, human tumors have lower pH values (~6.0/6.5) because of an elevated rate of glycolysis [275, 276]. pH-sensitive bonds have been developed for the coupling of PEG to liposomes [277] (Figure 1). pH-sensitive liposomes achieved a higher concentration of cargo in the cytoplasm and nucleus than non-pH-sensitive PEGylated liposomes* in vitro *and allowed faster intratumoral content release *in vivo* [278, 279]. In addition to tumor sensitivity, pH sensitive groups can potentiate the efficacy of targeted drug-loaded liposomes. 

Folate-targeting of daunorubicin-loaded liposomes by incorporation of a pH-sensitive folate-PEG-cholesterol hemisuccinate (CHEMS) conjugate combined tumor targeting and increased drug release at the tumor site with improved chemotherapeutic activity over untargeted liposomes [280]. Similarly, untargeted cisplatin-loaded liposomes or EGFR-targeted gemcitabine-loaded liposomes incorporating CHEMS had superior antitumor activity over untargeted drug-loaded liposomes or free drugs [281, 282]. Obata et al. used a glutamic acid-based zwitterionic lipid (1,5-dihexadecyl N,N-diglutamyl-lysyl-L-glutamate) as titratable lipid for doxorubicin delivery [283]. These liposomes showed a charge inversion from negative to positive at acidic pH with endosomal escape leading to higher doxorubicin delivery in the cytoplasm and higher toxicity *in vitro* over conventional liposomes. This resulted in superior antitumor activity *in vivo*. Biswas et al. developed a new pH-sensitive DSPE-PEG-hydrazone-PEG2000 conjugate for attachment of ligands to the liposome surface [284]. In their work, the cell penetrating peptide (TATp) was unmasked after PEG release at acidic pH allowing efficient cellular uptake.

Recently, three new approaches for generation of pH sensitivity have been reported. First, by electrostatic adsorption of negatively charged carboxyl-modified gold nanoparticles to the surface of cationic liposomes (egg dipalmitoylphosphatidylcholine/DOTAP 9 : 1 weight ratio) at pH 7 (pKa of 5 for the carboxylic group) [285]. Authors reported detachment of gold nanoparticles at acidic pH due to protonation of the carboxyl groups and speculated that a similar strategy could be applied with negative charged liposomes and amine-modified gold nanoparticles. Second, a platform for finely tuned pH-induced PEG release was introduced using phenyl-substituted-vinyl-ether-(PIVE)-PEG lipid conjugates [286]. Liposomes containing PIVE showed pH-induced dePEGylation and content release at acidic pH whereas they were stable at physiological pH. Third, ligand unmasking by acidic pH-induced membrane reorganization has been introduced as a reversible ligand-masking strategy. Sofou and coworkers developed a new platform for pH-triggered liposomal drug delivery [287, 288]. The rationale for their design involves the increased permeability at the boundaries between lipid domains [289]. Using lipid pairs of phosphatidic acid as a titrable headgroup and phosphatidylcholine as the colipid headgroup with mismatched hydrophobic chain lengths (dipalmitoyl and distearoyl) they demonstrated that formation of heterogeneous domains in PEGylated liposomes containing 5% of cholesterol allowed faster pH-dependent content release than liposomes with matched chains [288]. They showed a pH-dependent membrane transition due to the protonation of phosphatidylserine at lower pH in cholesterol-rich membranes, with protonation favoring their homologous interaction, leading to the formation of DSPS (1,2-distearoyl-sn-glycero-3[phosphor-L-serine]) lipid domains. PEG-lipid conjugates of matching hydrophobic anchor (DSPE-PEG) also segregated to these domains at acidic pH, whereas no redistribution of unmatched chain DPPE-PEG was in evidence [290]. The liposomes contained a ligand (biotin or an anti-HER2 peptide) harbored by an unmatched lipid (DPPE) which was masked by PEG at physiological pH but freed from PEG shielding at acidic pH after formation of the lipid heterogeneities. Application of this strategy to doxorubicin-loaded PEGylated (DSPE-PEG2000) liposomes harboring an HER2-specific peptide led to pH-dependent doxorubicin release *in vitro* and superior tumor growth inhibition than did untargeted vesicles or targeted vesicles devoid of pH-responsiveness [291].

### 5.2. MMP-Sensitive PEG Release

Hatakeyama and coworkers introduced coupling of PEG to DOPE by an MMP-cleavable linker, since MMPs are overexpressed in the tumor environment [292, 293]. Transfection efficiency *in vitro* was correlated with MMP levels and lipoplexes prepared with a MMP-responsive PEG-lipid conjugate showed tumor-specific transgene expression when compared to PEGylated lipoplexes with higher transgene expression for the same quantity of delivered lipoplexes. To enhance tumor targeting, Zhu et al. combined an MMP2-sensitive PEG-lipid conjugate with antibody targeting and an intracellular penetrating moiety (TaT peptide) [294] combining long circulation by PEGylation, tumor targeting via antinuclear antibody 2C5, and selective internalization by tumor cells through MMP-2 triggered exposure of TaT peptide.

### 5.3. Redox-Sensitive PEG Release

Tumor cells have a higher concentration of reductases than the extracellular environment or normal cells and this feature has promoted the use of disulfide linkers both for the design of reduction-sensitive PEG-lipid conjugates and crosslinked nanoparticles, since the linker is stable in the circulation and normal tissues but reduced in the tumor cells [295, 296]. Goldenbogen et al. developed a versatile reduction-sensitive conjugate for targeted delivery [297]. Biotin was conjugated to a lipid anchor via a disulfide linker to prepare biotin-decorated liposomes; conjugation of streptavidin-HER2 monoclonal antibody allowed superior cellular uptake of doxorubicin *in vitro* over untargeted liposomes. Interestingly, less intracellular doxorubicin was detected after incubation with unsensitive HER2 targeted doxorubicin-loaded liposomes than reduction-sensitive targeted liposomes, further demonstrating the need for multifunctional liposomes. A combination of enhanced uptake and reduction-sensitivity was also done using reduction-detachable PEG and TAT [298]. Cleavage of DOPE-S-S-PEG5000 allowed unmasking of DOPE-PEG1600-TAT and superior uptake of calcein *in vitro* over uncleavable TAT-modified liposomes together with stability in the presence of serum. Reduction-sensitive liposomes have also been used for gene delivery and a linear correlation between intracellular glutathione content and transfection efficiency has been recently demonstrated [299].

## 6. Intracellular Delivery

**Figure 4:**
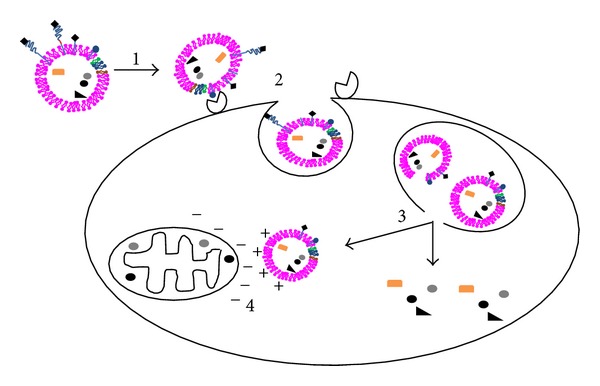
Strategies for intracellular delivery. Steps for intracellular delivery: (1) Stimuli-sensitive activation/unmasking of internalization moiety, (2) Cancer cell-specific endocytosis, (3) Endosomal escape and/or therapeutic agent release after activation of fusogenic peptides or lipids, (4) Binding to the highly negative mitochondrial outer membrane for mitochondria targeting. Legends are the same as in Figure 1.

Internalization of anticancer drugs by cancer cells in tumors was shown to be a barrier to be overcome for cancer therapy [98, 101]. The use of internalization modifications at the liposomal surface or exposed after release of a PEG corona in the tumor-environment for active transport into cells and even subcellular delivery increased therapeutic activity [7, 17, 96, 300]. The influence of lipid composition on drug release and internalization, endosomal escape strategies, and mitochondria targeting is discussed below (Figure 4).

### 6.1. Importance of Lipid Composition

The presence of cholesterol or rigid saturated lipids (DSPC, HSPC) stabilizes the liposomal membrane against liposomal dissociation by plasma proteins and limits drug leakage, and thus most drug-loaded liposomes include cholesterol in the lipid bilayer [45, 288, 301]. These lipids have high gel-to-liquid crystalline phase transition temperatures (55–58°C) compared to physiological temperature (37°C) which prevents coexistence of the two phases and contributes to improved drug pharmacokinetics [13, 45, 302]. In some studies, the couple sphingomyelin/cholesterol is used to further rigidify the membrane through hydrogen bonding [303]. However, cholesterol inclusion can decrease drug loading. Indeed, paclitaxel loading decreased form 99.3% at a 5% molar content of cholesterol to 66.5% at 17% cholesterol content and 6.2% at a 37% molar content as a result of the hindered drug penetration in the increasingly rigid lipid bilayer [304]. The lipid composition is also important for the choice of the PEG-lipid conjugate used for PEGylation. Indeed, Kusumoto et al. reported a 10-fold higher transfection using liposomes armed with an endosomal-escape peptide (IFN7) harboring cholesteryl-PEG2000 over DSPE-PEG2000 [305]. The superior endosomal escape of liposomes prepared with the former was attributed to the higher fluidity of cholesterol over DSPE, a superior fluidity favoring interaction with endosomal membranes and the resulting endosomal escape and transfection efficiency. Hydrophobicity was also shown to be a determinant for the design of smart multifunctional nanocarriers. Hansen et al. compared UV-triggered TaT peptide-mediated liposome internalization with a 16 or 12 carbons lipid anchor [306]. In addition to better internalization, liposomes with a C16 anchor were less prone to aggregation than those with a C12 anchor. The authors suggested the more hydrophobic alkyl chain favored liposomal insertion and the burial of the TaT peptide in a PEG-loop for the best UV-responsiveness and stability in cell culture media with bovine serum albumin.

Insertion of negatively charged lipids such as cardiolipin has been used to increase the retention of positively charged drugs in liposomes [45]. This was recently well illustrated for the preparation of mitoxantrone liposomes (mitoxantrone-complexed liposomes) by electrostatic complexation between anionic cardiolipin-based liposomes and cationic mitoxantrone [307]. While loading efficiencies of 95% were obtained with anionic liposomes using cardiolipin (CA), cholesteryl hemisuccinate (CHEMS), egg L-*α*-phosphatidylglycerol (PG), or L-*α*-phosphatidylserine (PS), only 3.8% loading was achieved with neutral liposomes. The therapeutic activity of the different anionic liposomal mitoxantrone preparations was in good agreement with release of mitoxantrone, that is, with the mitoxantrone release *in vitro* after heparin treatment. CHEMS liposomes had the lowest retention capacity and had virtually no impact on the survival of peritoneal carcinoma-bearing mice, and both PS and PG liposomes had intermediate mitoxantrone retention and exhibited higher therapeutic activity than free drug, albeit still inferior to that of CA liposomes capable of the highest mitoxantrone retention *in vitro*. Inclusion of anionic lipids should be coupled with PEGylation, since a negative charge directs liposomes to liver and spleen [308].

Lipid composition is also determinant for stimuli-responsive drug release. Goldenbogen et al. reported no calcein release from disulfide conjugated dipalmitoylphosphatidylcholine liposomes after treatment with a reducing agent, whereas reduction-induced release was observed from liposomes including 55% of unsaturated dioleoylphosphatidylethanolamine [297]. Note that Candiani et al. also incorporated DOPE in the lipid composition for bioreducible gene delivery, stressing the importance of DOPE as a helper lipid for membrane destabilization [299]. Increased permeability for thermosensitive drug release has been addressed by inclusion of 1-palmitoyl-2-hydroxy-sn-glycero-3-phosphocholine (P-lyso-PC) due to its tendency to form micelles and allow therapeutic efficacy *in vivo* of doxorubicin-loaded thermosensitive liposomes [309]. Nevertheless, the pharmacokinetics after administration in dogs was more similar to free doxorubicin than Doxil, which demonstrates the need to further optimize the lipid composition. Although liposomal cisplatin with 80% hydrogenated soy phosphatidylcholine (HSPC) showed increased cisplatin accumulation in preclinical tumors over free drug [21], this did not translate into therapeutic activity in patients [310, 311]. Absence of clinical activity was correlated with a lack of detectable released drug in the serum of treated patients, revealing the need for a balance between modifying the free drug pharmacokinetics for improved biodistribution to the diseased site and bioavilability [96]. PEGylation is required for enhanced blood residency and therapeutic efficacy, but postinsertion of DSPE-PEG6000 into preformulated siRNA lipoplexes was reported to induce siRNA release *in vitro* [312] and was nicely overcome by the use of cholesterol grafted siRNA for increased retention in liposomes. The combination of cellular uptake and targeting using a cholesterol-siRNA conjugate and cyclic RGD peptide allowed luciferase silencing in a B16F10-luc 2 experimental lung metastasis model, validating this new system [313].

### 6.2. Cell Penetrating Peptides

Cell penetrating peptides (CPPs) are amphiphatic peptides, usually cationic, either derived from viruses or synthetic that are able to improve the cellular internalization of the attached cargo [314] (Figure 4). The most frequently used CPPs are the TaT peptide derived from the transcription-transactivating protein of human immunodeficiency virus type 1 and synthetic polyarginine [315, 316]. TaT peptide is a powerful internalization moiety. However its endocytosis lacks cell-specificity and TaT peptide exposure at the liposome surface can lead to MPS elimination after opsonin binding as well [317]. For Tat-mediated internalization only in the tumor environment, masking strategies have been proposed. This concept was proved by Kale and Torchilin using masked TaT peptide surface-functionalized lipoplexes prepared with a plasmid coding for GFP (DSPE-PEG1000-TAT) by a pH-sensitive PEG corona (DSPE-hydrazone-PEG2000), leading to higher transgene expression in tumor tissue after intratumoral injection of pH-sensitive formulations [318]. Kuai et al. masked TaT peptide at the liposome surface (TAT-PEG2000-DSPE) by a reduction-sensitive PEG corona (PEG5000-S-S-DSPE) to take advantage of the higher concentration of reductive enzymes in tumors [319]. This allowed higher tumor accumulation and less liver uptake than unmasked Tat peptide-modified liposomes after intravenous administration. 

More recently, UV-triggered CPPs have been proposed [306]. They added a CPP through incorporation of a TaT peptide-lipid conjugate with two lipid anchors, a TaT peptide-PEG2000-DSPE conjugate linked to a less stable single chain hydrophobic group of 12 or 16 carbons via a UV-cleavable linker. They demonstrated a UV-dependent internalization of liposomes (a 15-fold increase in cellular adhesion and internalization only after irradiation), not observed with an uncleavable linker, that reached levels comparable to DSPE-PEG2000-TaT peptide liposomes. For the same purpose of cell-type selective CPP-mediated uptake, Kibria et al. functionalized liposomes with either RGD peptide or the tumor endothelial cell-specific peptide KYND and the octaarginine CPP and showed synergy of the combination of targeting peptide and cell penetrating peptide for liposome uptake *in vitro* with higher cell selectivity [320]. The same group later demonstrated superior antitumor activity of doxorubicin-loaded liposomes harboring both the tumor endothelial cell-specific peptide NGR and the cell penetrating peptide tetraarginine over untargeted liposomes or single-modified doxorubicin-loaded liposomes [183]. Presentation of octaarginine at the surface of bleomycin-loaded liposomes increased apoptosis induction in tumors and tumor growth inhibition over bleomycin-loaded liposomes devoid of the CPP [321]. Superior tumor growth inhibition was evidenced over untargeted RTN (receptor-targeted nanocomplexes, RTN) using lipopolyplexes decorated with an integrin-targeting peptide for delivery of pDNA encoding IL-2 and IL-12 to promote antitumor immunity [322, 323]. In their study, the complexes were optimized for disassembly in the target cell [323, 324]. The PEG-lipid conjugates used had an esterase-cleavable bond for endosomal escape and the integrin-targeting peptide was coupled to the polycation used for pDNA condensation by a linker cleavable by both cathepsin B and along with furin for intracellular release of the nucleic acid and high transfection efficiency.

In addition to enhancing cellular uptake, TaT peptide conjugation allowed crossing of the blood brain barrier in *in vitro* models and increased drug delivery of doxorubicin-loaded liposomes, resulting in prolonged survival of orthotopic glioma-bearing animals after intravenous administration [325]. 

### 6.3. Endosomal Escape

After the endocytosis, the cargo is transferred from endosomes (pH 6.5–6) to lysosomes (pH < 5) [326] in which enzymatic degradation occurs. Although PEGylation is required for extended blood circulation and tumor accumulation [7], this modification decreases cellular uptake and further increases endosomal degradation of the cargo, thereby reducing its activity [327, 328]. These conflicting properties of PEG have been referred to as the “PEG dilemma” [292]. The decreased endosomal pH has been exploited as a means to escape degradation using either fusogenic lipids or peptides which destabilize membranes after conformational activation at low pH, amines protonable at acidic pH for endosome swelling and rupture by a buffer effect [329–338] (Figure 4). The peptides used are either derived from viruses such as TATp from Human Immunodeficiency Virus [339], IFN7 from the haemagglutinin of influenza virus [340], or artificial peptides like GALA [341]. Inclusion of these peptides leads to superior intracellular drug accumulation and resulting in higher cytotoxicity than liposomes devoid of endosomolysis properties. As a new approach, Kullberg et al. attached the pore-forming protein listeriolysin O to HER2-targeted bleomycin-loaded liposomes, resulting in a higher toxicity *in vitro* over targeted bleomycin-loaded liposomes without listeriolysin O [342]. 

### 6.4. Mitochondrial Targeting

Effective treatment of cancer faces problems due to limited drug penetration and drug resistance [343–345]. Since resistance to antineoplastic agents induced cell death is frequently associated with altered mitochondrial function and/or altered expression of mitochondrial regulators of apoptosis [300, 343], subcellular accumulation of anticancer drugs in mitochondria can give a therapeutic advantage and has been exploited [300, 346] (Figure 4).

Mitochondria targeting of epirubicin-loaded liposomes by inclusion of the positively charged electrolyte dequalinium increased their cytotoxicity *in vitro* and antitumor activity *in vivo* over untargeted liposomes [347]. Hatakeyama and coworkers developed a Mito-Porter multifunctional envelope-type nanodevice (MEND) nanocarrier with sequential activation of essential functions necessary for mitochondria delivery [292, 346, 348]. These formulations have a “programmed packaging”; their surface is functionalized with a targeting moiety (transferrin or antibody), a PEG-lipid conjugate for long blood circulation; and a PEG-lipid bond that is cleaved in the tumor environment by matrix metalloproteinases leading to exposure of a CPP (octaarginine, tetraarginine) for tumor-selective endocytosis. Once inside the cell, a fusogenic peptide (KALA or GALA) allows endosomal escape of positively charged liposomes by membrane fusion, the positive charge favoring their interaction with the largely negative outer mitochondrial membrane, and finally the fusogenic lipid DOPE allows internalization of the cargo by the mitochondria [346]. Although complex, such nanocarriers are produced in GMP conditions warranting their clinical evaluation [348]. 

Instead of using one moiety for each step of intracellular targeting, Zhang and coworkers designed a smart, pH-responsive lipid (1,5-dioctadecyl-L-glutamyl-2-histidyl-hexahydroxybenzoic acid, HHG2C_18_) [349]. The liposomes generated are negatively charged at physiological pH and have a sharp charge inversion at acidic pH (from −22.9 mV at pH 7.4 to +6.3 mV at pH 6.5) for tumor-selective uptake. After uptake, hexahydrobenzoic acid is released by cleavage of the *β*-carboxylic acid linker in the endosomes leading to exposure of histidine and the endosomal escape of positively charged liposomes electrostatically targeted to the outer mitochondrial membrane. Liposomes containing the HHG2C_18_ lipid and encapsulating the anticancer drug Temsirorimus showed higher renal cancer tumor growth inhibition than free drug or nonresponsive liposomes. Targeting of topotecan-loaded PEGylated liposomes to mitochondria by inclusion of dequalinium, a lipophilic cation with a delocalized charge center that is attracted by the mitochondrial transmembrane potential [350], showed higher therapeutic efficacy than untargeted drug-loaded liposomes or free drug in two animal tumor models. 

In another study [351], postinsertion of the mitochondriotropic dye Rh123-PEG2000-DSPE conjugate into PEGylated liposomes permitted their mitochondrial accumulation and increased the toxicity of paclitaxel-loaded liposomes over untargeted liposomes or free drug. This result is in line with the activation of the intrinsic apoptosis pathway by paclitaxel [352]. Although these modifications lead to superior cytotoxicity, the lack of cancer cell specificity can decrease their therapeutic index. To address this challenge, the same authors modified paclitaxel-loaded liposomes with a mitochondriotropic lipid (triphenylphosphonium, TPP) TPP-PEG-PE conjugate [353]. While the PEGylation of liposomes leads to their extravasation into the tumor by the EPR effect, TPP modification allowed superior therapeutic activity of mitochondria-targeted liposomes since more drug was intracellularly available. Malhi et al. developed “mitocancerotropic” doxorubicin-loaded liposomes combining tumor targeting by folic acid and mitochondriotropism by TPP [354]. Dual-targeted liposomes led to higher doxorubicin accumulation in mitochondria and superior toxicity than single-targeted doxorubicin-loaded liposomes, thus warranting further evaluation of this strategy. 

## 7. Remote-Controlled Payload Release

To achieve release of the therapeutic agent at the tumor site, several strategies have been explored including ultrasound-triggered, photo-triggered, thermotriggered content release after controlled destabilization of the lipid bilayer (Figure 2).

### 7.1. Ultrasonication

Ultrasound-induced membrane permeabilization has been used for external stimuli-triggered drug release form liposomes by thermal or nonthermal effects (reviewed in [355]). Using PEGylated cisplatin-loaded liposomes, a 70% drug release after external ultrasound heating and a 2.7-fold increase in drug content occured *in vivo* whereas only 3% cisplatin was released without ultrasound exposure, leading to the superior therapeutic activity of the formulation in ultrasound-treated mice [356]. A correlation between DSPE content in liposome membranes and sonosensitivity has also been reported [357].

### 7.2. Photo-Sensitive Release and Photodynamic Therapy

Photo-sensitive liposomal drug delivery relies on photodestabilization of the liposomal bilayer to release the encapsulated drug [358]. The liposomes used should be able to route the drug to the tumor and protect it from photodynamic damage [359]. Photodynamic therapy (PDT) consists of the destruction of tumors by light-activation of a photosensitizer, resulting in liberation of singlet oxygen that destroys the tumor by apoptosis, necrosis, or autophagy-induced cell death mechanisms [360]. Although the limited light diffusion of this approach has been challenged by coupling of a light source to diffusing tips to treat deeper tumors [361], the area of cell death induction is still restrained due to the short lifetime of singlet oxygen (nanoseconds) [360]. Moreover, as these agents are mainly hydrophobic, their administration is limited by their aggregation, and the technique is limited to detectable tumors due to the nonspecific photosensitization [360, 362, 363]. Liposomal delivery of photosensitizers would allow treatment of both primary tumors and metastases by enhanced uptake of the photosensitizer by tumor cells. Yavlovich et al. reported for the first time light-triggered release of doxorubicin from PEGylated liposomes after laser irradiation including 10% of the photopolymerizable diacetylene phospholipid (1,2bis-(tricosa-10, 12-diynoyl)-sn-glycero-3-phosphocholine, DC_8,9_PC) resulting in photo-triggered cell killing *in vitro* [359]. The encapsulation of zinc tetraphenylporphyrin into PEGylated, folate-targeted liposomes improved its uptake and cytotoxicity after irradiation compared to untargeted liposomes *in vitro* [364]. Bovis et al. compared the pharmacokinetics of m-THPC [5,10,15,20-tetra-(m-hydroxyphenyl)chlorin] administered either in its clinically approved ethanol/propylene glycol formulation (Foscan) or in PEGylated liposomes [363]. Formulation of m-THPC in liposomes decreased its blood clearance and decreased skin photosensitivity compared to Foscan. Furthermore, m-THPC showed superior tumor accumulation and higher tumor necrosis than Foscan supporting its preclinical evaluation. Using another m-THPC un-PEGylated liposomal formulation (dipalmitoylphosphatidylcholine/dipalmitoylphosphatidylglycerol, 9 : 1 molar ratio) Lasalle et al. stressed the importance of optimization of the delay between photosensitizer administration and irradiation [365]. Indeed, while no increase in survival of mammary carcinoma-bearing mice was observed compared to control for 1 h and 3 h drug-light intervals, 6 h and 15 h intervals cured 79% and 63% of mice, respectively.

### 7.3. Thermoresponsive Preparations

While lipids with high transition temperatures (above 55°C) are required for blood stability and to decrease blood leakage, inclusion of lipids with transition temperatures closer to physiological body temperature (40–45°C) allows induction of drug release after external localized heating [45]. Inclusion of low transition temperature lipids is a strategy used in tumor therapy for more than 30 years since the pioneering study of Weinstein et al. who used dipalmitoylphosphatidylcholine [366]. Doxorubicin-loaded liposomes containing 2% of poly [2-(2-ethoxy)ethoxyethyl vinyl ether (EOEOVE)], (transition temperature 40°C) exhibited a rapid doxorubicin release after heating to 45°C with limited release at 37°C, and allowed tumor growth suppression only after heating [367]. Interestingly, in their study thermoresponsiveness of poly (EOEOVE) liposomes was improved by coinclusion of DSPE-PEG5000 in the liposome formulation and revealed an advantage of multifunctional liposome PEGylation. Encapsulation of the doxorubicin analog, epirubicin into PEGylated thermoresponsive liposomes increased blood residency and tumor accumulation over unresponsive liposomes or free drug, resulting in a 20% higher tumor growth inhibition in animals treated with thermoresponsive liposomes over unresponsive epirubicin-loaded liposomes [368]. 

Paasonen et al. used gold-nanoparticles as “energy collectors” to lower the threshold energy required to induce photo-sensitive drug release [369]. After heat transfer from gold nanoparticles to lipids promoting liquid crystal-to-gel phase transition, a UV-induced liberation of the model compound calcein was evidenced with virtually no release without irradiation. Magnetic fluid hyperthermia involves heat transfer from magnetic particles after exposure to a magnetic field that results in localized elevation of temperature and induction of cell death [370]. To improve the selectivity, doxorubicin thermo-responsive liposomes coloaded with doxorubicin and magnetic nanoparticles were armed with folic acid and resulted in improved cytotoxicity *in vitro *over nonresponsive liposomes or untargeted thermo-responsive doxorubicin-loaded liposomes [371]. Intra-tumoral injection of anti-HER2 immunoliposomes containing magnetite followed by alternate magnetic field heating promoted iron retention in tumors in a HER2-specific manner 48 h after injection [372]. A 3-fold higher iron content was detected in HER2-overexpressing BT474 breast cancer xenografts over low HER2-expressing SKOV3 ovarian cancer xenografts, and magnetite retention in BT474 xenografts correlated with stable tumor regression [372]. In line with these studies, conjugation of HER2 antibody to thermo-sensitive doxorubicin-loaded liposomes improved the doxorubicin-mediated toxicity over controls [373]. 

Boron capture neutron therapy relies on delivery of ^10^B boron followed by *γ*-irradiation and capture of neutrons by ^10^B, leading to the production of toxic *α*-particles, ^4^H and ^7^Li for cell death induction [374]. Maruyama encapsulated ^10^B into PEGylated transferrin-armed liposomes for targeted delivery to colon carcinoma xenografts, this led to higher ^10^B tumor accumulation compared to the free isotope or untargeted liposomes and resulted in superior therapeutic efficacy after irradiation over free isotope or untargeted ^10^B liposomes [36]. Lastly, the group led by Miyata reported a 3.6-fold higher ^10^B tumor concentration in orthotopic gliomas after intratumoral convection-enhanced delivery using PEGylated transferrin armed liposomes over untargeted liposomes with a lower retention in normal brains [375]. Superior therapeutic activity was observed against intracranial gliomas after intravenous injection of transferrin-targeted liposomes encapsulating sodium borocaptate over untargeted ones after neutron irradiation [376].

## 8. Theranostic Liposomes

Simultaneous therapy and diagnosis following codelivery of therapeutic and imaging agents, theranostic, are determinant for the development of personalized medicine since it would allow clinicians to detect and characterize lesions and rapidly evaluate tumor response and modify treatment accordingly (increase dose, stop treatment, or use an alternate drug) [377–379]. Indeed, liposomes are currently widely used for diagnosis (see recent reviews) [380–382].

Kenny et al. designed PEGylated liposome-entrapped siRNA nanoparticles (LEsiRNA) loaded with gadolinium (III) for magnetic resonance imaging, siRNA against the apoptosis inhibitor survivin for tumor therapy, and labeled with DOPE-rhodamine for fluorescence detection [383]. Accumulation of LEsiRNA in ovarian cancer xenografts after intravenous injection was demonstrated by MRI and confirmed *post mortem* in tumor biopsies by fluorescence with *in vivo* survivin silencing and tumor weight reduction. Gd-labeled, doxorubicin-loaded thermo-responsive liposomes allowed detection of both tumor imaging by MRI and tumor regression after localized heating [384]. Note that to retain thermoresponsiveness after Gd-labeling a new Gd-chelate-dendron-based lipid was included in the lipid bilayer instead of a standard Gd-lipid conjugate to decrease Gd-lipid content to enhance thermosensitivity. 

The use of magnetic resonance imaging (MRI) to allow both tumor visualization and temperature feedback for imaging-guided thermo-responsive drug delivery showed improved therapy of the image-guided, thermallyinduced drug release [385, 386]. Labeling of prednisolone-labeled liposomes did not decrease its therapeutic activity, allowed evaluation of *in vivo* drug biodistribution and response monitoring simultaneously, with MRI signal detection 1 week after injection [387]. To combine the advantages of three imaging modalities (optical imaging, CT imaging, and MRI), Li et al. and Mitchell et al. developed liposomes labeled with a fluorophore tracer, with ^99m^Tc, ^111^In or ^64^Cu, and Gd [388, 389]. Since most facilities do not possess all the imaging equipment, this system would allow a more flexible followup of therapeutic activity by optical imaging, while in depth studies would use CT or MRI without the need of administration of another imaging agent. Spatially controlled thermallyinduced drug release was achieved with MRI-guided high intensity focused ultrasound heating of the targeted tumor region resulting in deep tumor penetration of doxorubicin-loaded thermo-sensitive liposomes, coloading of liposomes with doxorubicin and gadolinium allowing tumor visualization and therapy [385, 386, 390]. 

The contrast agent used for the preparation of theranostic siRNA liposomes must be chosen with care. Mikhaylova et al. reported nonspecific protein downregulation *in vitro* after incorporation of gadolinium of Magnevist into COX-2 (cyclooxygenase 2) siRNA-loaded liposomes, while COX-2 silencing without nonspecific downregulation was detected with liposomes coloaded with COX-2 siRNA and Feridex [391]. Targeting drug-loaded liposomes, in addition to enhancing their therapeutic activity, enhances tumor detection and response monitoring when they are coloaded with an imaging agent. Addition of transferrin to ^10^B plus iodine contrast agent coloaded liposomes allowed a 3.6-fold higher ^10^B concentration in tumor tissues over untargeted coloaded liposomes [375]. The selective retention of transferrin-targeted formulations led to better tumor detection 72 h after administration of liposomes, a period during which the signal from untargeted liposomes had washed out, thus combining monitoring of drug delivery and tumor response with boron neutron capture therapy [375]. Combined delivery of Gd and doxorubicin in liposomes targeted with a neural cell adhesion molecule-specific peptide allowed higher concentration of doxorubicin in tumor tissues correlated with increased tumor growth inhibition over untargeted coloaded liposomes together with better visualization of tumors by MRI [392]. Targeting of iron oxide and doxorubicin coloaded liposomes to pancreatic tumors by conjugation of an antimesothelin antibody improved the antitumor activity and tumor signal enhancement over untargeted liposomes [393]. Folate targeting of doxorubicin-loaded liposomes encapsulating iron oxide resulted in superior tumor growth inhibition of liver cancer tumors than the standard formulation Doxil and simultaneously allowed tumor imaging by MRI with higher sensitivity than the commercial contrast agent, Resovist [394].

## 9. Conclusions

In addition to the need for extended blood circulation and stimuli-controlled extravasation to the tumor's niche, multifunctional liposomal nanocarriers must target at least one hallmark of cancer (aberrant cell growth, drug resistance, sustained angiogenesis, and tissue invasion) for enhancement of tumor therapy and/or diagnosis. As described throughout the paper, this requires coordinated action of stealth, targeting, and internalizing moieties to achieve intracellular delivery to cancer cells in tumors. Moreover, combined targeting of tumor cells and related neoangiogenesis is becoming a focus of research that allows destruction of both primary and distant tumor nodules. However, targeted therapies rely on ligands presented by a few types of tumors and must face up to the fact of the heterogeneity of tumor cells and their surface markers [175, 395, 396]. A possible direction may be the coupling of ligands of different natures (antibody, protein, peptides and chimiokine, hormone analogs) to target at least two tumor cell populations for relapse-free cancer therapy and more sensitive malignant lesion detection.

## Figures and Tables

**Table 1:** Examples of multifunctional liposomal nanocarriers.

Encapsulated agent	Targeting ligand	Development stage	References
Doxorubicin	None	Approved (Doxil/Caelyx)	[13]
Vincristine	None	Approved (Marqibo)	[14]
Paclitaxel	None	Approved (Lipusu)	[15]
Cytarabine and daunorubicin	None	Phase I (CPX-351)	[24]
Irinotecan and floxuridine	None	Phase I (CPX-1)	[25]
PKN3 siRNA	None	Phase I (Atu-027)	[26]
Irinotecan	None	Phase I (NL CPT-11)	[27]
Doxorubicin	Stomach cancer-specific anti-GAH mAb	Phase I (MCC-465)	[28]
Oxaliplatin	Transferrin	Phase II (MBP-426)	[29]
Liposomal p53 DNA and docetaxel	Anti-Transferrin receptor scFv	Phase I (SGT53-01)	[30]
Doxorubicin	Thermoresponsive liposomes	Phase III (ThermoDox)	[31]
Doxorubicin	Cancer-specific 2C5 mAb	preclinical	[32]
Doxorubicin	Anti-CD22 mAb	preclinical	[33]
Paclitaxel	Anti-HER2 mAb	preclinical	[34]
Vincristine	mBAFF	preclinical	[35]
Oxaliplatin	Transferrin	preclinical	[36]
Daunorubicin	Transferrin and mannose	preclinical	[37]
Vinorelbine	NSCLC-specific peptide	preclinical	[38]
Doxorubicin	Metastasis-specific peptide	preclinical	[39]
Doxorubicin	MMP-2/9 detachable PEG	preclinical	[40]
Irinotecan	Folic acid	preclinical	[41]
Doxorubicin	Estrone	preclinical	[42]
Etoposide	Chondroitin sulfate	preclinical	[43]

**Table 2:** Examples of ligands used for targeting of liposomal nanocarriers.

Type of ligand	Ligand	Target	Reference(s)
Antibody	Anti-HER2	HER2 receptor overexpressed by cancer cells	[34, 98, 100]
	Anti-CD19	CD19 overexpressed in B cell Lymphoma	[101]
	Nucleosome-specific 2C5 mAb	Cancer cells surface-bound nucleosomes	[32, 102]

Protein	Transferrin	Transferrin receptor overexpressed by cancer cells	[36, 103]
	Interleukin 13 (IL-13)	IL-13 receptor overexpressed in human gliomas	[104]

Peptide	Octreotide	Somatostatin receptor type 2 overexpressed by cancer cells	[105, 106]
	LHRH-derived peptide	LHRH receptors overabundant on cancer cells	[107]
	Arg-Gly-Asp (RGD)	*α*V*β*3overexpressed by endothelial tumor cells	[108–110]

Small molecule	Folate	Folate receptor on cancer cells	[41, 111]
	Estrone	Estrogen receptors overexpressed in ovarian and breast cancers	[42, 112]
	Anisamide	Sigma receptors overexpressed by cancer cells	[113]

Sugar	Mannose	Dendritic cells and macrophages to induce an immune response	[114, 115]
	Lactose	Asialoglycoprotein receptors overexpressed by hepatocellular carcinomas	[116]

HER2: human epidermal growth factor receptor 2, mAb: monoclonal antibody, LHRH: luteinizing hormone releasing hormone.
